# Comparison of Giemsa and Hemacolor Staining Reveals Systematic Differences in Avian Leukocyte Profiles

**DOI:** 10.1002/ece3.74540

**Published:** 2026-10-22

**Authors:** Joanna Skwarska

**Affiliations:** ^1^ Department of Experimental Zoology and Evolutionary Biology, Faculty of Biology and Environmental Protection University of Lodz Łódź Poland

**Keywords:** avian leukocyte identification, blood smear staining, Giemsa stain, Hemacolor

## Abstract

Accurate identification of avian leukocyte fractions from blood smears depends strongly on the staining method. This study provides a comparative evaluation of Giemsa and Hemacolor staining, integrating qualitative and quantitative assessments based on paired samples from 39 Great Tits *Parus major* . Leukocyte profiles derived from Hemacolor‐stained smears showed significantly higher heterophil counts and approximately 40% higher H:L ratios, whereas Giemsa‐stained smears yielded higher lymphocyte proportions; eosinophil counts did not differ significantly between methods. These quantitative differences were consistent with microscopic characteristics: Hemacolor generated a lighter field with distinctly defined heterophilic granules, while Giemsa provided stronger nuclear contrast and facilitated lymphocyte identification. The consistent directional differences observed between staining methods indicate a systematic bias in leukocyte assessment. Because the two methods emphasize different cellular features, they produce non‐interchangeable leukocyte profiles with implications for ecological and physiological interpretation. Accordingly, staining protocols should be explicitly reported, and a single method should be applied consistently within a dataset.

## Graphical abstract

This study compares two commonly used staining methods (Giemsa and Hemacolor) and shows that they produce systematically different leukocyte profiles in birds. These differences have important implications for the interpretation of physiological stress and immune parameters in ecological studies.

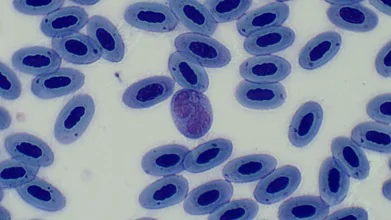

## 1 Introduction

For decades, analysis of blood parameters has been used as an indicator of health and physiological condition in living organisms, including birds (Campbell 1995; Davis et al. 2008). Common hematological indices in birds include hemoglobin concentration, erythrocyte count and mean corpuscular volume (MCV), hematocrit, and the number and relative proportions of leukocyte types, particularly the heterophil‐to‐lymphocyte ratio (H:L ratio) (Davis et al. 2008; Vinkler et al. 2010). Unlike mammalian blood, avian erythrocytes are nucleated, which precludes the use of standard automated hematology analyzers calibrated for anucleate mammalian red cells. Thus, automated staining/counting devices are not recommended for avian blood smears (Campbell 1995). Romanowsky‐type staining methods such as Giemsa and Hemacolor are commonly used in avian hematology. Both techniques are currently employed for avian blood smear staining but differ in staining characteristics, contrast, and visibility of cellular structures. Comparative evaluations of Romanowsky‐type staining methods have also been conducted in other vertebrates with nucleated erythrocytes (Arvan et al. 2025). These studies indicate that stain selection may influence the visualization of nuclear and cytoplasmic features and consequently affect hematological assessment, emphasizing the importance of methodological consistency in blood smear analysis.

Stained smears enable identification and differentiation of blood cells, morphological assessment, detection of parasites (e.g., *Plasmodium*) (Minichová et al. 2024), including those associated with haemosporidian infection parameters (Emmenegger and Bensch 2026), and estimation of leukocyte fractions, including the H:L ratio (Skwarska et al. 2019, 2022). Because the H:L ratio is often used to summarize leukocyte redistribution and is widely interpreted as a proxy of physiological condition and environmental stress, methodological factors affecting leukocyte identification may directly influence biological interpretation of results.

Bird‐derived physiological and hematological indicators are frequently applied in ecological studies addressing processes such as host–parasite interactions, disease dynamics, and environmental stress responses. For example, studies on avian‐mediated transmission of pathogens have highlighted the ecological relevance of data collected from wild birds (Wawrzyniak et al. 2025). Consequently, methodological inconsistencies in leukocyte assessment may propagate into broader ecological conclusions.

In hematological diagnostics, Hemacolor and modified Giemsa staining represent two widely used Romanowsky‐type techniques for preparing peripheral blood smears. Although both methods aim to enhance cytological detail and enable reliable differentiation of leukocyte populations, they differ in procedural characteristics, notably staining time, contrast intensity, and susceptibility to artifacts related to fixation, pH conditions, and reagent quality. These distinctions may influence the clarity of nuclear and cytoplasmic structures as well as the visibility of characteristic granules in granulocytic lineages.

The aim of this paper is to test whether the choice of staining method introduces systematic differences in leukocyte counts and the H:L ratios, and to evaluate whether these differences may bias ecological and physiological interpretations based on leukocyte‐derived indicators.

## 2 Materials and Methods

The research material was collected between 5 and 26 January 2024 as part of a long‐term project on the physiological condition of Great Tits *Parus major* in central Poland. The dataset comprised samples from 39 Great Tits (27 males and 12 females). Great Tits were selected because they are the focal species of a long‐term research program conducted by our research group and were also the most abundant species captured during the winter sampling period. The sample size was determined by the number of individuals captured during the study period. Because each individual provided paired blood smears analyzed using both staining methods, the paired design enabled direct within‐individual comparisons and increased statistical power. Birds were captured using standard mist nets (5 m in length) between 09:00 and 13:00. Each bird was ringed with an aluminum band with a unique identification number, and sex was determined. From each individual, two blood samples (approximately 5–10 μL) were drawn from the ulnar vein to prepare paired blood smears. One smear was stained using the Giemsa method and the other with Hemacolor.

Blood smears were stained using standard Giemsa and Hemacolor protocols. Detailed staining procedures are provided in Methods S1. Leukocyte identification was based on the morphological criteria described by Campbell (1995) and Canfield (1998); detailed identification criteria are provided in Methods S1. Stained smears were examined under oil immersion at 1000× magnification using an MBL‐320T light microscope (Mikrolab, Poland), and leukocyte counts were performed up to 100 cells per smear (Campbell 1995). All counts were conducted twice for each slide by a single observer (J.S.). To assess intra‐observer repeatability, intraclass correlation coefficients (ICC) were calculated based on the duplicate counts performed for each smear.

Because leukocyte proportions and H:L ratios did not meet the assumptions of normal distribution, nonparametric paired tests were applied. Differences between staining methods (Hemacolor vs. Giemsa) were evaluated using the Wilcoxon signed‐rank test, which accounts for both the direction and magnitude of paired differences. To verify the robustness of these results, the sign test was additionally performed as a conservative test assessing only the direction of change. All tests were two‐tailed. All statistical analyses were performed using Statistica 13 (TIBCO Software Inc., Palo Alto, CA, USA; TIBCO Software Inc. 2017).

Full individual leukocyte counts for all birds, including values for heterophils, lymphocytes, eosinophils, and the H:L ratio, are provided in Table S1.

## 3 Results

The results of the Wilcoxon signed‐rank test comparing mean leukocyte values between the two staining methods are presented in Table 1. Because the Wilcoxon test evaluates both the magnitude and direction of paired differences, significant results indicate that the observed shifts between staining methods were both consistent and sufficiently large across paired observations.

**TABLE 1:** Wilcoxon signed‐rank test and sign test results comparing mean leukocyte values between Hemacolor and Giemsa staining methods. Effect size (*r*) was calculated for the Wilcoxon signed‐rank test.

Variable	Hemacolor	Giemsa	Wilcoxon signed‐rank test			Effect size (*r*)	Sign test			*n*
	Mean		*T*	*Z*	*p*		Percentage of *V* < 0	*Z*	*p*	
Heterophil	29.64	15.90	19.00000	5.18	**< 0.000001**	**0.83**	5.13	5.44	**< 0.000001**	39
Lymphocyte	60.35	75.36	13.00000	5.188	**< 0.000001**	**0.84**	94.74	5.35	**< 0.000001**	38
Eosinophil	7.46	8.45	300.00000	1.26	0.209135	0.20	64.10	1.60	0.109135	39
H:L ratio	0.69	0.24	11.00000	5.29	**< 0.000001**	**0.85**	5.13	5.44	**< 0.000001**	39

*Note:* Bold values indicate statistically significant results (*p* < 0.05).

The sign test, which assesses only directional consistency, yielded the same qualitative conclusions (Table 1). Hemacolor smears showed significantly higher heterophil counts and the H:L ratios than Giemsa, whereas lymphocyte proportions were significantly higher in Giemsa‐stained slides. No significant differences were found for eosinophils. Both statistical tests indicated the same directional trends. Specifically, heterophil proportions were higher in Hemacolor‐stained smears in 37 of 39 individuals, whereas lymphocyte proportions were higher in Giemsa‐stained smears in 36 of 39 individuals (one individual showed identical values). Similarly, H:L ratios were higher in Hemacolor‐stained smears in 37 of 39 individuals. These results demonstrate that the observed differences were not driven by a few extreme observations but reflected a highly consistent shift across individuals. The biological relevance of these differences was further supported by large effect sizes for heterophils (*r* = 0.83), lymphocytes (*r* = 0.84), and the H:L ratio (*r* = 0.85), whereas eosinophils showed only a small effect size (*r* = 0.20) (Table 1). Intra‐observer repeatability was high for the principal leukocyte variables. Intraclass correlation coefficients ranged from 0.95 to 0.96 for heterophils, from 0.89 to 0.95 for lymphocytes, and from 0.85 to 0.95 for H:L ratios, indicating high agreement between replicate counts. Repeatability for eosinophils was lower (ICC = 0.42–0.60), likely owing to their relatively low abundance in the examined smears.

These differences correspond to key qualitative staining characteristics of both methods. Hemacolor provides clearer visualization of heterophilic granules, whereas Giemsa enhances nuclear contrast and facilitates lymphocyte identification. Thus, the choice of staining method introduces a systematic numerical bias that should be considered when interpreting leukocyte profiles and H:L‐based physiological indicators.

To illustrate these methodological differences, the table below provides a comparative overview of key diagnostic and technical parameters, including the color characteristics of individual leukocyte fractions, contrast properties, and the methods' usefulness for archival purposes (Table 2). Because certain leukocyte features—especially granule coloration in eosinophils, heterophils, and basophils—may vary subtly between the two staining techniques, a direct comparison helps highlight where Hemacolor offers rapid and consistent staining, while modified Giemsa provides a more traditional, detail‐enhancing chromatic profile (Table 2). Representative examples of blood smears illustrating staining differences are shown in Figure 1 and Figures S1–S4.

**TABLE 2:** Diagnostic and Morphological Features of Leukocyte Fractions Stained with Hemacolor and Giemsa.

Feature	Hemacolor	Giemsa
Overall field	Lighter palette; nuclei magenta/violet; cytoplasm blue‐gray	Darker palette; strong nuclear contrast (red‐violet to violet)
Heterophils	Distinct violet granules; segmented nucleus—easier to recognize than with Giemsa	Granules often pale violet, less conspicuous in dense areas; lobed nucleus
Eosinophils	Red to red‐brown granules; pale blue cytoplasm; lobed nucleus	Reddish to red‐brown granules; pale blue cytoplasm; nucleus often bluer/more distinct
Basophils	Dark violet to black granules that may obscure the light blue nucleus	Deep violet/black granules (may coalesce/dissolve in some alcohol‐soluble variants)
Lymphocytes	Blue‐gray homogeneous cytoplasm; high n:c; beware confusion with thrombocytes (clear/reticulated cytoplasm, occasional small red granules, tendency to clump)	Homogeneous light blue cytoplasm; high n:c (small/medium/large forms)
Monocytes	Blue‐gray, slightly granular cytoplasm; vacuoles possible; eccentric nucleus	Blue‐gray cytoplasm (often two zones) with vacuoles; irregular/bilobed nucleus

**FIGURE 1:**
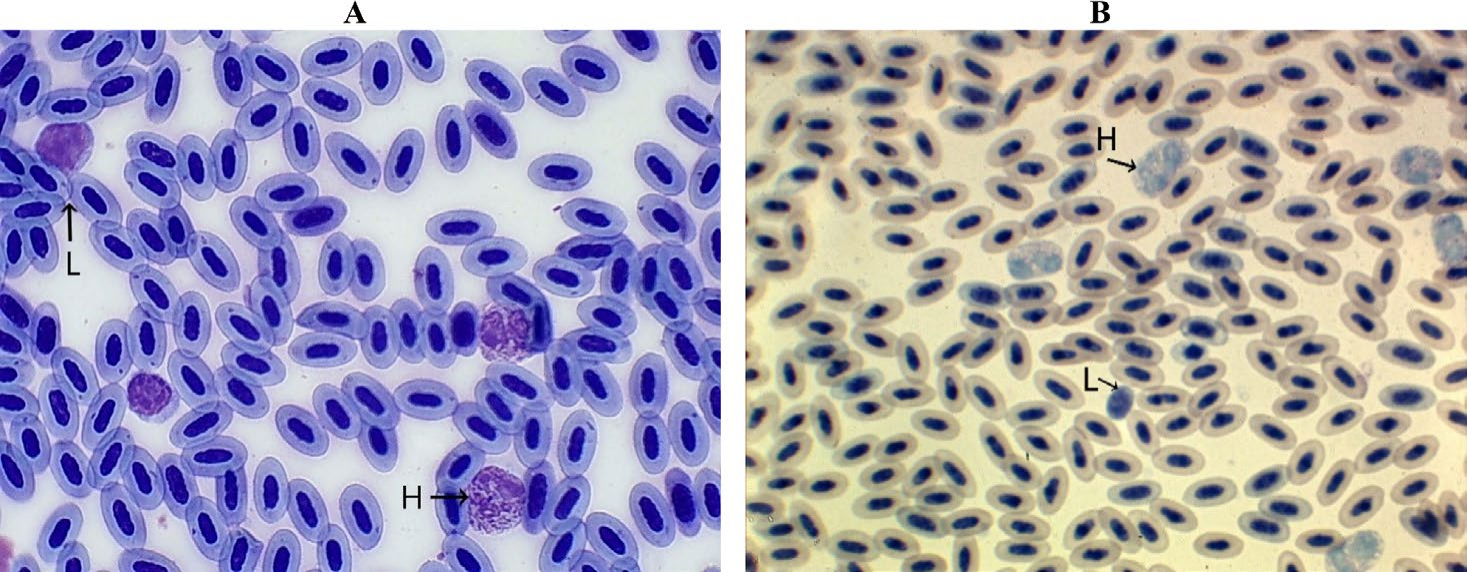
Representative blood smears stained with Hemacolor (A) and Giemsa (B). Arrows indicate representative heterophils (H) and lymphocytes (L), facilitating direct comparison of the staining characteristics of both leukocyte types between methods. Hemacolor staining provides clearer visualization of heterophilic granules, whereas Giemsa staining enhances nuclear contrast and facilitates lymphocyte identification.

Microscopic images of blood smears illustrating differences between Hemacolor and Giemsa staining are provided in Figures S1–S4. These images enable direct visual comparison of staining intensity, granule coloration, nuclear contrast, and overall morphological clarity in both methods, complementing the descriptive information summarized in Table S2.

## 4 Discussion

Because bird‐derived physiological indicators are frequently used in ecological studies addressing topics such as host–parasite interactions and disease dynamics, methodological inconsistencies in leukocyte assessment may propagate into broader ecological conclusions. The present study demonstrates that the choice of staining method can lead to consistent and systematic differences in leukocyte profiles derived from avian blood smears. These differences are not random but directionally consistent, suggesting that they reflect methodological bias rather than measurement noise.

Differences observed between Giemsa and Hemacolor staining can be explained by variation in staining characteristics, including contrast, chromatic intensity, and the visibility of cytoplasmic granules and nuclear structures. These differences in visualization can directly influence leukocyte classification and subsequent quantitative estimates. Such methodological effects have important implications for the interpretation of leukocyte‐based indices. In particular, the H:L ratio is widely used as a proxy for physiological stress and immune condition in ecological and evolutionary studies. If staining method systematically shifts the relative proportions of leukocyte types, the resulting H:L ratios may not be directly comparable across studies, populations, or time periods.

Importantly, the consistency of these differences across individuals observed in this study indicates a directional shift rather than random variation. This suggests that datasets generated using different staining methods may yield systematically different estimates of physiological condition, potentially leading to misinterpretation of ecological patterns. Such bias is especially critical in studies that compare populations, assess temporal trends, or synthesize results across multiple datasets. Differences attributed to environmental conditions, habitat quality, or life‐history variation may in part reflect methodological inconsistencies rather than true biological differences. This issue is particularly relevant in research areas relying on avian physiological indicators, including studies on host–parasite interactions, disease dynamics, and responses to environmental stress.

## 5 Conclusions

My results demonstrate that the choice of staining method is a significant and non‐neutral source of variation in avian leukocyte profiles. Hemacolor staining tends to yield higher heterophil proportions and the H:L ratios, whereas Giemsa staining results in relatively higher lymphocyte counts, indicating that these methods produce systematically different quantitative outcomes.

Because the H:L ratio is widely used as an indicator of physiological condition and environmental stress, such method‐dependent differences may influence ecological and evolutionary interpretations. Consequently, the H:L ratios derived from different staining methods should not be treated as directly comparable.

I emphasize that strict methodological consistency within studies is essential, and staining protocols should be explicitly reported to ensure transparency and comparability across datasets. Mixing staining methods within a single dataset should be avoided, as it may introduce artificial variation unrelated to biological processes.

## Author Contributions

**Joanna Skwarska:** conceptualization (equal), data curation (equal), formal analysis (equal), funding acquisition (equal), investigation (equal), methodology (equal), project administration (equal).

## Funding

The author has nothing to report.

## Ethics Statement

This study was approved by the Local Bioethical Commission for Experiments on Animals, Medical University in Lodz (No. 70/ŁB07/2015 and 11/ŁB07/2016) and the Regional Directorate for Environmental Protection (WPNII.6401.13.2016.MS, WPNII.6401.122.2016.KW2). Our team is based on wild animals in the field. In addition, we did not conduct manipulation experiments in this study. Therefore, the stipulations that applied to wild animals were respected by us, in accordance with ARRIVE guidelines (http://arriveguidelines.org). We can also confirm that all methods were performed in accordance with the relevant guidelines and regulations.

## Conflicts of Interest

The author declares no conflicts of interest.

## Supporting information


**Table S1:** Individual leukocyte counts and H:L ratios in Great Tits, based on two replicate counts per staining method (Hemacolor and Giemsa). H—heterophil, L—lymphocyte, E—eosinophil, HL—heterophil‐to‐lymphocyte ratio, x̄—mean value.

**Supplementary File S2:** Supplementary Methods, combined cytological and technical characteristics of avian blood smears stained using Giemsa and Hemacolor methods (Table S2), leukocyte identification criteria, and supplementary references.

**Figure S1:** Hemacolor‐stained blood smear showing a representative eosinophil. The cell is characterized by a lobed nucleus and distinct cytoplasmic granules visible against the background of nucleated avian erythrocytes. This image illustrates the typical morphology and staining characteristics of eosinophils in Hemacolor‐stained smears.
**Figure S2:** Hemacolor‐stained blood smear showing a lymphocyte (left) and a heterophil (right). The lymphocyte is characterized by a large, densely stained nucleus occupying most of the cell area and a narrow rim of cytoplasm. In contrast, the heterophil exhibits a segmented nucleus and numerous distinct cytoplasmic granules. This image illustrates the morphological differences between these two major leukocyte types and demonstrates the clear visualization of heterophil granules achieved with Hemacolor staining.
**Figure S3:** Hemacolor‐stained blood smear showing a heterophil (left) and an eosinophil (right). The heterophil is characterized by a segmented nucleus and numerous elongated cytoplasmic granules, whereas the eosinophil displays a lobed nucleus and more rounded, densely stained granules. The image illustrates morphological features used to distinguish these two granulocyte types and demonstrates the clear visualization of cytoplasmic granules obtained with Hemacolor staining.
**Figure S4:** Giemsa‐stained blood smear showing an eosinophil (top center) and a heterophil (bottom right) among nucleated avian erythrocytes. The image illustrates the typical morphology of these granulocyte types and the staining characteristics observed in Giemsa‐stained smears.

## Data Availability

All data generated or analyzed during this study are included in this published article (and its Supporting Information).
