# Supplementary material for: Comparison of Giemsa and Hemacolor Staining Reveals Systematic Differences in Avian Leukocyte Profiles
Source: Ecol Evol. 2026 Oct 22;16(10):e74540. doi: 10.1002/ece3.74540 (PMC13691185; doi:10.1002/ece3.74540)
Supplement: Supplementary file 1 [file ECE3-16-e74540-s001.docx]

Detailed staining procedures

Blood smears were prepared immediately after blood collection by spreading a small drop of blood into a thin, uniform layer on a clean glass slide. Particular care was taken to ensure appropriate smear thickness, as overly thick smears can impede stain penetration and obscure cellular structures, especially in nucleated avian blood cells (Bennett 1970; Campbell 1995). All smears were air-dried for at least 30 minutes prior to staining.

Giemsa staining

Air-dried blood smears were fixed by immersion in methanol for 3 minutes. After fixation, the slides were stained in Giemsa solution for 20 minutes (PanReac AppliChem; Thermo Fisher Scientific). Following staining, the slides were rinsed in tap water to remove excess stain and then air-dried.

Hemacolor® rapid staining

Blood smears were stained using the Hemacolor® kit (Merck), following the standard protocol. The staining procedure consisted of three steps: fixation in a methanol-based solution, staining with an eosin solution, and staining with an azur-based solution (**Hemacolor – Merck KGaA n.d.)**. Slides were immersed sequentially in each solution for short intervals according to the manufacturer's instructions.

Following staining, the slides were rinsed in phosphate buffer (pH 7.2) prepared using buffer tablets dissolved in distilled water. The buffer was prepared at least one day prior to staining to ensure stability. After rinsing, slides were air-dried.

Microscopic examination

After staining with either Giemsa or Hemacolor®, blood smears were examined under oil immersion at 1000× magnification. Leukocyte identification was based on morphological characteristics described in standard avian hematology references (Campbell 1995; Canfield 1998).

Notes on leukocyte identification

Distinguishing between leukocyte types was based on morphological criteria, including nuclear shape, cytoplasmic characteristics, and granule morphology. Heterophils typically exhibited elongated, rod-like granules, whereas eosinophils possessed more rounded and intensely stained granules. Lymphocytes were characterized by a high nucleus-to-cytoplasm ratio and homogeneous cytoplasm, while thrombocytes were distinguished by their tendency to aggregate and by cytoplasmic structure. Monocytes showed larger size, irregular nuclei, and vacuolated cytoplasm.

The table below summarizes key cytological and technical differences between Giemsa and Hemacolor® staining methods. It highlights staining-specific features that may influence leukocyte identification and contribute to method-dependent variation in leukocyte profiles.

Supplementary Table S2. Combined cytological and technical characteristics of avian blood smears stained using Giemsa and Hemacolor® methods

| Parameter / Cell Type | Giemsa | Hemacolor^®^ |
| --- | --- | --- |
| Nuclei | red to violet | purple to violet |
| Erythrocytes | Reddish | reddish |
| Thrombocytes | Violet | violet |
| Monocytes – cytoplasm | grey-blue | dove-blue |
| Lymphocyte identification | very good (nuclear detail stands out) | good |
| Lymphocytes – cytoplasm | Blue | blue-grey |
| Azurophilic granules | – | magenta |
| Heterophil identification | harder in dense smears (granules often pale violet) | easier (violet granules clearly defined) |
| Heterophilic granules | light violet granules | light violet granules |
| Eosinophils – granules | reddish to red-brown | red to red-brown |
| Basophils – granules | dark violet | dark violet to black |
| Time | ≈20–25 min | rapid (seconds; dip protocol) |
| Contrast | darker field; strong nuclear contrast; granules subtler in thick zones | lighter field; granules distinct |
| Artifacts sensitivity | pH/dilution/thickness strongly influence colours; stock care matters | occasional residues; buffer prep critical |
| Suitability / typical uses | broad cytology/cytogenetics; widely used historically | fast routine diagnostics |

**Supplementary References:**

**Bennett, G.F. 1970.** Simple techniques for making avian blood smears. *Canadian Journal of Zoology* **48**: 585–586. https://doi.org/10.1139/z70-098

**Campbell, T. W. 1995.** Avian Hematology and Cytology, 2nd edn. Iowa State University Press, Ames

**Canfield, P. J. 1998.** Comparative cell morphology in the peripheral blood film from exotic and native animals. *Australian Veterinary Journal* 76: 793–800

**Hemacolor – Merck KGaA. n.d.** Hemacolor^®^ rapid blood smear staining: Instructions. https://www.merckmillipore.com/deepweb/assets/sigmaaldrich/product/documents/291/741/111957-pl-mk.pdf

**PanReac AppliChem. n.d.** Giemsa stain, modified solution for clinical diagnosis: Instruction manual. https://www.itwreagents.com/itwreagents_files/info_points/IP-014/en_GB.pdf

**Thermo Fisher Scientific. n.d.** Giemsa stain: Instruction manual. https://documents.thermofisher.com/TFS-Assets/LSG/manuals/IFU246403.pdf
