# Supplementary material for: Comparison of Giemsa and Hemacolor Staining Reveals Systematic Differences in Avian Leukocyte Profiles
Source: Ecol Evol. 2026 Oct 22;16(10):e74540. doi: 10.1002/ece3.74540 (PMC13691185; doi:10.1002/ece3.74540)
Supplement: Supplementary file 2 [file ECE3-16-e74540-s002.docx]

Figure S1. Hemacolor^®^-stained blood smear showing a representative eosinophil. The cell is characterized by a lobed nucleus and distinct cytoplasmic granules visible against the background of nucleated avian erythrocytes. This image illustrates the typical morphology and staining characteristics of eosinophils in Hemacolor^®^-stained smears.

Figure S2. Hemacolor^®^-stained blood smear showing a lymphocyte (left) and a heterophil (right). The lymphocyte is characterized by a large, densely stained nucleus occupying most of the cell area and a narrow rim of cytoplasm. In contrast, the heterophil exhibits a segmented nucleus and numerous distinct cytoplasmic granules. This image illustrates the morphological differences between these two major leukocyte types and demonstrates the clear visualization of heterophil granules achieved with Hemacolor^®^ staining.

Figure S3. Hemacolor^®^-stained blood smear showing a heterophil (left) and an eosinophil (right). The heterophil is characterized by a segmented nucleus and numerous elongated cytoplasmic granules, whereas the eosinophil displays a lobed nucleus and more rounded, densely stained granules. The image illustrates morphological features used to distinguish these two granulocyte types and demonstrates the clear visualization of cytoplasmic granules obtained with Hemacolor^®^ staining.

Figure S4. Giemsa-stained blood smear showing an eosinophil (top center) and a heterophil (bottom right) among nucleated avian erythrocytes. The image illustrates the typical morphology of these granulocyte types and the staining characteristics observed in Giemsa-stained smears.
