# Supplementary material for: Comparison of Giemsa and Hemacolor Staining Reveals Systematic Differences in Avian Leukocyte Profiles
Source: Ecol Evol. 2026 Oct 22;16(10):e74540. doi: 10.1002/ece3.74540 (PMC13691185; doi:10.1002/ece3.74540)
Supplement: Supplementary file 3 [file ECE3-16-e74540-s003.docx]

Supplementary Table S1. Individual leukocyte counts and H:L ratios in Great Tits, based on two replicate counts per staining method (Hemacolor® and Giemsa). H – heterophil, L – lymphocyte, E – eosinophil, HL – heterophil-to-lymphocyte ratio, x̄ – mean value.

|  | Haemacolor® | | | | | | | | | | | | Giemsa | | | | | | | | | | | |
| --- | --- | --- | --- | --- | --- | --- | --- | --- | --- | --- | --- | --- | --- | --- | --- | --- | --- | --- | --- | --- | --- | --- | --- | --- |
| Bird ID | H_1_–H | H_2_–H | x̄H–H | L_1_–H | L_2_–H | x̄L–H | H:L_1_–H | H:L_2_–H | x̄H:L –H | E_1_–H | E_2_–H | x̄E–H | H_1_–G | H_2_–G | x̄H–G | L_1_–G | L_2_–G | x̄L–G | H:L_1_–G | H:L_2_–G | x̄H:L–G | E_1_–G | E_2_–G | x̄E– G |
| 1 | 38 | 29 | 33.5 | 49 | 55 | 52 | 0.76 | 0.53 | 0.65 | 13 | 15 | 14 | 12 | 15 | 13.5 | 65 | 78 | 71.5 | 0.18 | 0.19 | 0.19 | 23 | 7 | 15 |
| 2 | 15 | 18 | 16.5 | 72 | 70 | 71 | 0.21 | 0.26 | 0.23 | 11 | 9 | 10 | 6 | 5 | 5.5 | 87 | 91 | 89 | 0.07 | 0.05 | 0.06 | 5 | 4 | 4.5 |
| 3 | 33 | 32 | 32.5 | 55 | 50 | 52.5 | 0.60 | 0.64 | 0.62 | 9 | 13 | 11 | 18 | 18 | 18 | 75 | 73 | 74 | 0.24 | 0.25 | 0.24 | 7 | 9 | 8 |
| 4 | 28 | 25 | 26.5 | 45 | 58 | 51.5 | 0.62 | 0.43 | 0.53 | 21 | 16 | 18.5 | 7 | 9 | 8 | 81 | 82 | 81.5 | 0.09 | 0.11 | 0.10 | 12 | 9 | 10.5 |
| 5 | 18 | 18 | 18 | 76 | 76 | 76 | 0.24 | 0.24 | 0.24 | 5 | 3 | 4 | 5 | 5 | 5 | 88 | 90 | 89 | 0.06 | 0.05 | 0.06 | 7 | 5 | 6 |
| 6 | 14 | 12 | 13 | 76 | 81 | 78.5 | 0.18 | 0.15 | 0.17 | 9 | 4 | 6.5 | 6 | 7 | 6.5 | 72 | 83 | 77.5 | 0.08 | 0.08 | 0.08 | 20 | 10 | 15 |
| 7 | 30 | 30 | 30 | 65 | 65 | 65 | 0.46 | 0.46 | 0.46 | 4 | 3 | 3.5 | 17 | 13 | 15 | 76 | 86 | 81 | 0.22 | 0.15 | 0.19 | 7 | 4 | 5.5 |
| 8 | 56 | 55 | 55.5 | 41 | 40 | 40.5 | 1.37 | 1.36 | 1.37 | 2 | 5 | 3.5 | 22 | 21 | 21.5 | 76 | 70 | 73 | 0.29 | 0.30 | 0.29 | 2 | 9 | 5.5 |
| 9 | 17 | 20 | 18.5 | 67 | 65 | 66 | 0.25 | 0.31 | 0.28 | 11 | 13 | 12 | 10 | 14 | 12 | 79 | 74 | 76.5 | 0.13 | 0.19 | 0.16 | 11 | 12 | 11.5 |
| 10 | 30 | 30 | 30 | 65 | 58 | 61.5 | 0.46 | 0.52 | 0.49 | 4 | 6 | 5 | 14 | 9 | 11.5 | 81 | 75 | 78 | 0.17 | 0.12 | 0.15 | 5 | 16 | 10.5 |
| 11 | 42 | 40 | 41 | 50 | 52 | 51 | 0.84 | 0.77 | 0.80 | 5 | 7 | 6 | 28 | 25 | 26.5 | 62 | 64 | 63 | 0.45 | 0.39 | 0.42 | 10 | 11 | 10.5 |
| 12 | 27 | 29 | 28 | 47 | 48 | 47.5 | 0.57 | 0.60 | 0.59 | 24 | 15 | 19.5 | 15 | 18 | 16.5 | 70 | 71 | 70.5 | 0.21 | 0.25 | 0.23 | 15 | 10 | 12.5 |
| 13 | 3 | 6 | 4.5 | 91 | 89 | 90 | 0.03 | 0.07 | 0.05 | 1 | 4 | 2.5 | 11 | 13 | 12 | 77 | 80 | 78.5 | 0.14 | 0.16 | 0.15 | 12 | 7 | 9.5 |
| 14 | 85 | 67 | 76 | 10 | 14 | 12 | 8.50 | 4.79 | 6.64 | 4 | 19 | 11.5 | 37 | 36 | 36.5 | 45 | 54 | 49.5 | 0.82 | 0.67 | 0.74 | 18 | 10 | 14 |
| 15 | 33 | 27 | 30 | 43 | 53 | 48 | 0.77 | 0.51 | 0.64 | 16 | 17 | 16.5 | 19 | 17 | 18 | 75 | 75 | 75 | 0.25 | 0.23 | 0.24 | 6 | 8 | 7 |
| 16 | 43 | 40 | 41.5 | 51 | 52 | 51.5 | 0.84 | 0.77 | 0.81 | 4 | 4 | 4 | 37 | 33 | 35 | 52 | 51 | 51.5 | 0.71 | 0.65 | 0.68 | 10 | 16 | 13 |
| 17 | 7 | 8 | 7.5 | 84 | 90 | 87 | 0.08 | 0.09 | 0.09 | 8 | 0 | 4 | 1 | 6 | 3.5 | 98 | 90 | 94 | 0.01 | 0.07 | 0.04 | 0 | 4 | 2 |
| 18 | 12 | 15 | 13.5 | 80 | 79 | 79.5 | 0.15 | 0.19 | 0.17 | 6 | 3 | 4.5 | 8 | 6 | 7 | 83 | 92 | 87.5 | 0.10 | 0.06 | 0.08 | 9 | 2 | 5.5 |
| 19 | 11 | 12 | 11.5 | 74 | 81 | 77.5 | 0.15 | 0.15 | 0.15 | 8 | 3 | 5.5 | 9 | 6 | 7.5 | 79 | 80 | 79.5 | 0.11 | 0.07 | 0.09 | 14 | 14 | 14 |
| 20 | 22 | 24 | 23 | 70 | 67 | 68.5 | 0.31 | 0.38 | 0.34 | 5 | 8 | 6.5 | 17 | 17 | 17 | 79 | 79 | 79 | 0.21 | 0.21 | 0.21 | 4 | 4 | 4 |
| 21 | 31 | 25 | 28 | 50 | 50 | 50 | 0.62 | 0.50 | 0.56 | 15 | 19 | 17 | 16 | 15 | 15.5 | 69 | 76 | 72.5 | 0.23 | 0.20 | 0.21 | 15 | 9 | 12 |
| 22 | 21 | 18 | 19.5 | 71 | 76 | 73.5 | 0.30 | 0.24 | 0.27 | 4 | 4 | 4 | 7 | 6 | 6.5 | 90 | 87 | 88.5 | 0.08 | 0.07 | 0.07 | 3 | 7 | 5 |
| 23 | 32 | 29 | 30.5 | 59 | 63 | 61 | 0.54 | 0.46 | 0.50 | 7 | 2 | 4.5 | 17 | 18 | 17.5 | 63 | 72 | 67.5 | 0.27 | 0.25 | 0.26 | 18 | 10 | 14 |
| 24 | 42 | 46 | 44 | 52 | 49 | 50.5 | 0.81 | 0.94 | 0.87 | 6 | 2 | 4 | 27 | 29 | 28 | 68 | 65 | 66.5 | 0.40 | 0.45 | 0.42 | 3 | 6 | 4.5 |
| 25 | 45 | 41 | 43 | 42 | 46 | 44 | 1.07 | 0.89 | 0.98 | 12 | 11 | 11.5 | 18 | 20 | 19 | 71 | 72 | 71.5 | 0.25 | 0.28 | 0.27 | 11 | 8 | 9.5 |
| 26 | 27 | 29 | 28 | 59 | 58 | 58.5 | 0.46 | 0.50 | 0.48 | 11 | 6 | 8.5 | 10 | 17 | 13.5 | 71 | 75 | 73 | 0.14 | 0.23 | 0.18 | 19 | 8 | 13.5 |
| 27 | 44 | 47 | 45.5 | 52 | 48 | 50 | 0.85 | 0.98 | 0.91 | 3 | 5 | 4 | 13 | 20 | 16.5 | 83 | 77 | 80 | 0.16 | 0.26 | 0.21 | 4 | 3 | 3.5 |
| 28 | 40 | 40 | 40 | 57 | 48 | 52.5 | 0.70 | 0.83 | 0.77 | 3 | 10 | 6.5 | 15 | 13 | 14 | 72 | 83 | 77.5 | 0.21 | 0.16 | 0.18 | 13 | 3 | 8 |
| 29 | 25 | 28 | 26.5 | 71 | 67 | 69 | 0.35 | 0.42 | 0.38 | 3 | 3 | 3 | 10 | 8 | 9 | 79 | 83 | 81 | 0.13 | 0.10 | 0.11 | 6 | 9 | 7.5 |
| 30 | 22 | 22 | 22 | 67 | 57 | 62 | 0.33 | 0.39 | 0.36 | 7 | 16 | 11.5 | 33 | 27 | 30 | 64 | 66 | 65 | 0.52 | 0.41 | 0.46 | 1 | 1 | 1 |
| 31 | 14 | 14 | 14 | 76 | 81 | 78.5 | 0.18 | 0.17 | 0.18 | 7 | 1 | 4 | 4 | 3 | 3.5 | 91 | 96 | 93.5 | 0.04 | 0.03 | 0.04 | 5 | 5 | 2 |
| 32 | 56 | 48 | 52 | 39 | 44 | 41.5 | 1.44 | 1.09 | 1.26 | 5 | 7 | 6 | 38 | 38 | 38 | 57 | 62 | 59.5 | 0.67 | 0.61 | 0.64 | 5 | 0 | 2.5 |
| 33 | 20 | 23 | 21.5 | 79 | 76 | 77.5 | 0.25 | 0.30 | 0.28 | 1 | 1 | 1 | 11 | 14 | 12.5 | 85 | 83 | 84 | 0.13 | 0.17 | 0.15 | 3 | 3 | 3 |
| 34 | 31 | 28 | 29.5 | 63 | 57 | 60 | 0.49 | 0.49 | 0.49 | 5 | 9 | 7 | 5 | 7 | 6 | 82 | 80 | 81 | 0.06 | 0.09 | 0.07 | 13 | 13 | 13 |
| 35 | 19 | 17 | 18 | 76 | 77 | 76.5 | 0.25 | 0.22 | 0.23 | 4 | 5 | 4.5 | 7 | 6 | 6.5 | 86 | 91 | 88.5 | 0.08 | 0.07 | 0.07 | 7 | 3 | 5 |
| 36 | 51 | 44 | 47.5 | 41 | 49 | 45 | 1.24 | 0.90 | 1.07 | 7 | 5 | 6 | 45 | 39 | 42 | 46 | 52 | 49 | 0.98 | 0.75 | 0.86 | 6 | 9 | 7.5 |
| 37 | 56 | 55 | 55.5 | 36 | 38 | 37 | 1.56 | 1.45 | 1.50 | 4 | 5 | 4.5 | 26 | 22 | 24 | 57 | 64 | 60.5 | 0.46 | 0.34 | 0.40 | 17 | 14 | 15.5 |
| 38 | 16 | 20 | 18 | 66 | 70 | 68 | 0.24 | 0.29 | 0.26 | 14 | 8 | 11 | 11 | 8 | 9.5 | 81 | 87 | 84 | 0.14 | 0.09 | 0.11 | 8 | 5 | 6.5 |
| 39 | 20 | 25 | 22.5 | 75 | 68 | 71.5 | 0.27 | 0.37 | 0.32 | 3 | 5 | 4 | 11 | 14 | 12.5 | 75 | 79 | 77 | 0.15 | 0.18 | 0.16 | 11 | 7 | 9 |
